# Refining multivariate disease phenotypes for high chip heritability

**DOI:** 10.1186/1755-8794-8-S3-S3

**Published:** 2015-09-23

**Authors:** Jiangwen Sun, Henry R Kranzler, Jinbo Bi

**Affiliations:** 1 Department of Computer Science and Engineering, University of Connecticut, 371 Fairfield Way, U-4155, Storrs, CT, 06269, USA; 2 Treatment Research Center, University of Pennsylvania Perelman School of Medicine, 3900 Chestnut Street Philadelphia, PA, 19104, USA

**Keywords:** phenotype-genotype association analysis, chip heritability, quadratic optimization, heritable component analysis

## Abstract

**Background:**

Statistical genetics shows that the success of both genetic association studies and genomic prediction methods is positively associated with the heritability of the trait used in the analysis. Identifying highly heritable components of a complex disease can thus enhance genetic studies of the disease. Existing heritable component analysis methods use data from related individuals to compute linearly-combined traits to maximize heritability. Recent advances in acquiring genome-wide markers have enhanced heritability estimation using genotypic data from apparently unrelated individuals, which is referred to as the chip heritability. Novel statistical models are thus needed to identify disease components (subtypes) with high chip heritability.

**Methods:**

We propose an optimization approach to identify highly heritable components of a complex disease as a function of multiple clinical variables. The heritability of the components is estimated directly from unrelated individuals using their genome-wide single nucleotide polymorphisms. The proposed approach can also model the fixed effects due to covariates, such as age and race, so that the derived traits have high chip heritability after correcting for fixed effects. A new sequential quadratic programming algorithm is developed to efficiently solve the proposed optimization problem.

**Results:**

The proposed algorithm was validated both in simulations and the analysis of a real-world dataset that was aggregated from genetic studies of cocaine, opoid, and alcohol dependence. Simulation studies demonstrated that the proposed approach could identify the hypothesized component from multiple synthesized features. A case study on cocaine dependence (CD) identified a quantitative trait that achieved chip heritability of 0.86 estimated using a cross-validation process. This quantitative trait corresponded to the likelihood of an individual's membership in a CD subtype. Clinical analysis showed that the subtype enclosed individuals who reported heavy use of cocaine but few withdrawal symptoms.

**Conclusions:**

Extensive experiments on both synthetic and real-world data demonstrate the effectiveness of the proposed approach as a means to find meaningful disease components with high chip heritability.

## Introduction

Identifying genetic variation that underlies complex diseases has important implications in medicine. To date, genome-wide association studies (GWAS) have had limited success in dissecting the genetic etiology of complex diseases. For instance, very few associations identified for substance use disorders at a genome-wide significant level have been replicated [1-3]. Complex disorders are often characterized by multiple disease indicators. For example, to diagnose whether a patient has a lifetime drug dependence disorder, clinicians interview the patient to understand his or her drug use behaviors, the negative consequences of the drug use, the treatment history and other co-occuring medical conditions. All of these clinical variables are used to arrive at a diagnosis of dependence on a certain drug [4]. There is substantial variation in these variables in the disease population, and these variables also present different levels of heritability, i.e., some are more genetically influenced than others. This phenotypic heterogeneity diminishes evidence of genetic association. Statistical genetics also shows that the success of most gene discovery studies is positively associated with the heritability of the trait used in the association analysis [5]. Hence, identifying more homogeneous and highly heritable components of a complex disease could enhance the association analysis.

The ability to translate genotype information into a quantitative prediction of disease phenotypes is important for precision medicine [6]. Genomic prediction methods that predict a phenotype based on genome-wide single nucleotide polymorphisms (SNPs) may provide a suitable analytic tool [7,8]. These methods expand the traditional single-marker-regression-based GWAS model for detecting few variants of large effect to multi-marker predictive models with many variants of small effect. The predictive ability of genomic prediction methods relies on several factors, especially trait heritability [9]. If we identify higly heritable components of a complex disease, it could also improve the utility of genomic prediction methods to predict subtypes (defined by the components) of the disease.

Because the success of both association analysis and genomic prediction is dependent on the trait heritability, heritability can be a valid target for refining multivariate disease phenotypes. The narrow-sense heritability *h*^2 ^is defined by the percentage of phenotypic variance that is due to additive genetic effects [10]. The broad-sense heritability *H*^2 ^is defined as the overall genetic contribution to the phenotypic variation. The heritability of a quantitative trait is commonly estimated from related individuals in pedigrees. Recent advances in acquiring dense genome-wide genetic markers have enhanced heritability estimation from apparently unrelated individuals using their genome-wide SNPs. The SNP-based heritability, often referred to as the chip heritability, is defined as the portion of the phenotypic variation that can be explained by the genotyped genetic markers [11]. It has been argued that estimating *h*^2 ^from unrelated individuals has an advantage over traditional pedigree-based methods because the estimated chip *h*^2 ^corresponds only to the causal-variant heritability that is tagged by the genotyped SNPs [8,12].

Phenotype refinement is an important but underdeveloped genetics research area. Unsupervised cluster analysis or latent class analysis has been commonly used to partition a study population into subgroups based on clinical variables [13-18]. This approach can create subgroups of individuals that differ in clinical symptoms and features, but may have limited utility in genetic analysis. Because genetic data are not used during the creation of the subgroups, the resultant subtypes (subgroups) are not guaranteed to have high heritability, and hence may not be informative for genetic association.

More relevant to this present work, a number of prior methods identify the principal components of clinical data that are heritable, and characterize the components by linear combinations of clinical variables [19-23]. Thus, these methods are often called heritable component analysis. All existing methods decompose the variance of clinical data into two components: the variance due to additive genetic effects estimated from pedigrees; and the variance due to other effects (residuals). Then, they solve a generalized eigen-decomposition problem to identify the linear combination of the clinical variables that maximizes the ratio of additive-genetic variance versus the residual variance, thus leading to high heritability of the resultant linearly combined trait. Nearly all of these methods use pedigree-based heritability estimation (an exception is [23]), and all assume a genetic model that is based on a single causal variant, an assumption that is commonly violated for complex diseases.

Although the latest heritable component analysis method [23] is effective and computationally efficient, a fundamental question is how much heritability of the derived trait can be explained by the genotyped SNPs. Because GWAS and genomic predictions mainly utilize the genotyped SNPs, the utility of the derived trait may be limited by a low chip heritability. Thus, novel statistical models are needed to directly target high chip heritability. In this paper, we propose an approach to identify the components of a multivariate disease phenotype that maximizes the chip *h*^2^. To estimate the chip heritability of a given trait, the latest methods use the restricted maximum likelihood (REML) method, which assumes that the trait follows a mixed effect model with random genetic effects, and fixed effects due to covariates, such as age, sex and race [8,12]. To identify a trait of high chip *h*^2^, we need to solve the inverse problem of (chip) heritability estimation. In other words, we now search for a trait (e.g., a linearly-combined trait) so that its chip heritability is high when estimated using the REML method. Directly solving the inverse problem leads to a quadratic optimization problem that can be optimized efficiently via a sequential quadratic programming algorithm. We validated the proposed approach in simulations as well as in the analysis of a real-world dataset that was aggregated from genetic studies of cocaine, opioid, and alcohol dependence. Our experimental results demonstrated the effectiveness and generalizability of the proposed approach.

## Methods

### The proposed statistical model

Given a set of *n *subjects, we denote their trait values of a quantitative trait *y *by a vector **y **of length *n*. We use a matrix **Z***_n×m _*to represent their standardized genotypic data at *m *genetic markers, and **C***_n×p _*to represent their data on *p *covariates. The matrix **Z **is calculated from the genotypic data as follows. Let *f_j _*be the frequency of reference allele at the *j*-th genetic variant, *r_ij _*be the number of copies of reference allele that the *i*-th subject has at the *j*-th locus. The standardized geno-type *z_ij _*is calculated as $r_{ij}-\text{2}f_{j}/\sqrt{\text{2}f_{j}\left(\text{1}-f_{j}\right)}$[8]. The chip heritability estimation method assumes the following mixed-effect linear model [8,12] that characterizes how a phenotype is related to genotypes and covariates:

(1) y=Cβ+Zu+ε,

where ***ε ***is a vector of length *n*, which specifies residual effects. In Eq.(1), all covariates create fixed effects (fixed ***β***) on the phenotype whereas genetic effects are random (random **u**). Assume that **u **and ***ε ***follow Gaussian distributions: **u **~ *N *(**0**, **I${\sigma_{e}}^{\text{2}}$**) and ***ε **~ N *(**0**, **I${\sigma_{u}}^{\text{2}}$**). Then, the covariance of **y **between individuals, denoted by **Ω***_n×n_*, can be calculated as:

(2) $$\text{Ω}=\text{Z}{\text{Z}}^{T}{\sigma_{u}}^{2}+\text{I}{\sigma_{e}}^{2}$$

Let ${\sigma_{g}}^{\text{2}}$ be the phenotypic variance attributable to all of the *m *genetic causal variants. Then, we have $\sigma_{g}^{2}=m\sigma_{u}^{2}$. Let **G **= **ZZ***^T ^/m*, which is referred to as the genetic relationship matrix (GRM) among subjects determined by the causal variants. Then Eq. (2) can be re-written as:

(3) $$\text{Ω}=G{\sigma_{g}}^{\text{2}}+I{\sigma_{e}}^{2}$$

where${\sigma_{g}}^{\text{2}}$ and${\sigma_{e}}^{\text{2}}$ can be estimated by the REML method [24,11]. The chip her-itability estimated on the *m *causal variants is computed as${h_{p}}^{2}={\sigma_{g}}^{2}/{\sigma_{e}}^{2}$, where ${\sigma_{p}}^{2}={\sigma_{g}}^{2}+{\sigma_{e}}^{2}$ is the total phenotypic variance. Because the causal variants of *y *are usually unknown for a trait, recent research has proposed to estimate a GRM using genome-wide SNPs [8,12].

The main idea of REML for estimating the variance components is to first eliminate the fixed effect due to covariates from the observed values of *y *and then estimate the variance components from the random effect part. The REML finds *n *basis vectors represented by columns of a matrix **L***_n×n_*. This matrix has two sub-matrices **L **= [**L**_1 _**L**_2_] with **L**_1 _of size *n × p *and **L**_2 _of size *n × *(*n − p*). The two sub-matrices satisfy $L_{1}^{T}C=I_{p\times p}$, and $L_{2}^{T}C=0$. Let$\tilde{\text{y}}={\text{L}}^{T}\text{y}$, ${\tilde{y}}_{1}={\text{L}}_{1}^{T}\text{y}$ and ${Ỹ}_{\text{2}}=L_{2}^{T}y$. It can be derived that **ỹ** follows the following multivariate Gaussian distribution given the multivariate Gaussian assumption of **y**:

$$\tilde{\text{y}}=\left[\begin{array}{c} {\text{L}}_{1}^{T}\text{y}\\ {\text{L}}_{2}^{T}\text{y} \end{array}\right]\sim N\left(\left[\begin{array}{c} \beta \\ 0 \end{array}\right]\right),\left(\left[\begin{array}{c} {\text{L}}_{1}^{T}\text{Ω}{\text{L}}_{1}\\ {\text{L}}_{2}^{T}\text{Ω}{\text{L}}_{1} \end{array}\right)\left(\begin{array}{c} {\text{L}}_{1}^{T}\text{Ω}{\text{L}}_{2}\\ L_{2}^{T}\text{Ω}{\text{L}}_{2} \end{array}\right]\right)$$

We have ${\tilde{y}}_{2}\sim N\left(0,{\text{L}}_{2}^{T}\text{Ω}{\text{L}}_{2}\right)$ and the conditional distribution:

$${\tilde{\text{y}}}_{1}|{\tilde{\text{y}}}_{2}\sim N\left(\beta +{\text{L}}_{1}^{T}\text{Ω}{\text{L}}_{2}{\left({\text{L}}_{2}^{T}\text{Ω}{\text{L}}_{2}\right)}^{-1}{\tilde{\text{y}}}_{2},{\left(C^{T}\text{Ω}C\right)}^{-1}\right).$$

Then, the log likelihood of **ỹ** can be decomposed into:

$$\ell \left(\sigma_{g}^{2},\sigma_{e}^{2};\tilde{\text{y}}\right)=\ell_{1}\left(\sigma_{g}^{2},\sigma_{e}^{2};{\tilde{\text{y}}}_{1}|{\tilde{\text{y}}}_{2}\right)+\ell_{2}\left(\sigma_{g}^{2},\sigma_{e}^{2};{\tilde{\text{y}}}_{2}\right),$$

Where ℓ_2 _is not a function of the fixed-effect parameter ***β***. The two variance components, i.e.,$\sigma_{g}^{2}$ and$\sigma_{e}^{2}$ can be estimated by maximizing *l*_2_, and there is no additional information in ℓ_1 _for estimating the variance components. Once $\sigma_{g}^{2}$ and $\sigma_{e}^{2}$ are estimated, a generalized least squares estimate of ***β ***can be obtained as:

$$\hat{\beta }={\tilde{\text{y}}}_{\text{1}}-{\text{L}}_{1}^{T}\text{Ω}{\text{L}}_{2}{\left({\text{L}}_{2}^{T}\text{Ω}{\text{L}}_{2}\right)}^{-1}{\tilde{\text{y}}}_{2}.$$

The second log likelihood component *ℓ*_2 _is calculated as (after removing constant):

$$\ell_{2}\left(\sigma_{g}^{2},\sigma_{e}^{2};{\tilde{\text{y}}}_{2}\right)=-\frac{1}{2}\left(\text{ln}|{\text{L}}_{2}^{T}\text{Ω}{\text{L}}_{2}|+\;{\tilde{\text{y}}}_{2}^{T}{\left({\text{L}}_{2}^{T}\text{Ω}{\text{L}}_{2}\right)}^{-1}{\tilde{\text{y}}}_{2}\right).$$

It has been shown in an early work [25] that when $L_{1}^{T}C=I_{p\times p}$ and $L_{2}^{T}C=0$, we have

$$\text{Ω}-\text{Ω}{\text{L}}_{2}{\left({\text{L}}_{2}^{T}\text{Ω}{\text{L}}_{2}\right)}^{-1}{\text{L}}_{2}^{T}\text{Ω}=C{\left(C^{T}{\text{Ω}}^{-1}C\right)}^{-1}C^{T}$$

Substituting these equations into the calculation of ℓ_2 _yields:

(4) $$\ell_{2}\left(\sigma_{g}^{2},\sigma_{e}^{2};{\text{y}}_{2}\right)=-\frac{1}{2}\left(\text{ln}|\text{Ω}|+\text{ln}|C^{T}{\text{Ω}}^{-1}C|{\tilde{\text{y}}}^{T}Py\right),$$

where $P={\text{Ω}}^{-1}-{\text{Ω}}^{-1}C{\left(C^{T}{\text{Ω}}^{-1}C\right)}^{-1}C^{T}{\text{Ω}}^{-1}$ and ***β ***can be obtained by:

(5) $$\beta ={\left(C^{T}{\text{Ω}}^{-1}C\right)}^{-1}C^{T}{\text{Ω}}^{-1}y$$

Given data on **y**, **C **and **Z**, $\sigma_{g}^{2}$ and $\sigma_{e}^{2}$ are obtained by maximizing the log like-lihood of observing the trait values $\ell \left(\sigma_{g}^{2},\sigma_{e}^{2};y\right)$ which corresponds to maximizing $\ell_{2}\left(\sigma_{g}^{2},\sigma_{e}^{2};{\tilde{\text{y}}}_{2}\right)$[11]. The chip heritability of a trait *y *is computed using the resultant optimal $\sigma_{g}^{2}$ and $\sigma_{e}^{2}$.

In our study, however, we solve the inverse problem of the above estimation model. A definitive quantitative trait *y *is not known beforehand but needs to be derived from a set of known clinical variables. Let **X***_n×d _*be the data matrix of *d *clinical variables **x **for the same *n *subjects as in **Z**. A trait *y *is defined by a linear function of *y *= **w^┬^x **where **w **is the vector of combination coefficients. Correspondingly, the trait values **y **= **Xw**. Unlike the heritability estimation process that finds the best values of $\sigma_{g}^{2}$ and $\sigma_{e}^{2}$ to maximize the likelihood of observing the values of *y*, the inverse problem searches for the best **w **so to form a trait **y **that maximizes the likelihood, (or equivalently the log likelihood $\ell \left(\sigma_{g}^{2},\sigma_{e}^{2};y,C,Z\right)$), of observing a large heritability, i.e., a large $\sigma_{g}^{2}$ but small $\sigma_{e}^{2}$. For simplicity and easy interpretation of the resultant model, here we only consider linear models, but the proposed method can be easily extended to construct non-linear models through kernel mapping [26].

Notice that the highest possible heritability of a trait *y *is 1 when $\sigma_{g}^{2}=1$ and $\sigma_{e}^{2}=0$. We hence propose to formulate an optimization problem, in which we search for the optimal **w **that maximizes the log likelihood $\ell \left(\sigma_{g}^{2},\sigma_{e}^{2};y,C,Z\right)$ (or equivalently, $\ell_{2}\left(\sigma_{g}^{2},\sigma_{e}^{2};{\tilde{\text{y}}}_{2},\right)$) of observing $\sigma_{g}^{2}=1$ and $\sigma_{e}^{2}=0$. According to Eq.(3), the covariance matrix **Ω **= **G **when $\sigma_{g}^{2}=1$ and $\sigma_{e}^{2}=0$. We substitute the values of these parameters into the log likelihood Eq.(4), and remove any constant terms. The resultant maximization problem is equivalent to the following minimization problem:

(6) $$\underset{W}{\text{min}}\;w^{T}\left(X^{T}PX\right)w$$

where **P **is calculated as:

(7) $$P=G^{-1}-G^{-1}C{\left(C^{T}G^{-1}C\right)}^{-1}C^{T}G^{-1}.$$

When $\sigma_{g}^{2}=1$ and $\sigma_{e}^{2}=0$, we have $\sigma_{p}^{2}=1$ because $\sigma_{p}^{2}=\sigma_{g}^{2}+\sigma_{e}^{2}$ This requires to impose a constraint to the optimization problem so that the total phenotypic variance that is due to either genetic or environmental effect should be scaled to1. An estimate of $\sigma_{p}^{2}$ can be obtained by calculating the sample variance after correcting for the covariate effects as ${\hat{\sigma }}_{p}^{2}=\frac{1}{n}{\left(Xw-C\beta \right)}^{T}\left(Xw-C\beta \right)$. Since ***β ***can be estimated according to Eq.(5), by substituting the ***β ***value, ${\hat{\sigma }}_{p}^{2}$ can be computed by

$${\hat{\sigma }}_{p}^{2}=\frac{1}{n}w^{T}X^{T}\text{(}J^{T}J\text{)}Xw$$

where $J=I-C{\left(C^{T}{\text{Ω}}^{-1}C\right)}^{-1}C^{T}{\text{Ω}}^{-1}$. To further simplify the notation, denote

(8) $$Q=\frac{J^{T}J}{n},$$

Then

$${\hat{\sigma }}_{p}^{2}=w^{T}\left(X^{T}QX\right)w.$$

Combining the objective function and the constraint together, the proposed optimization problem is formulated as:

(9) $$\begin{array}{c} \underset{w}{\text{min}}\;w^{T}\left(X^{T}PX\right)w,\\ \text{subject}\;\text{to}\;w^{T}\left(X^{T}QX\right)w=1. \end{array}$$

According to statistical learning theory [26], only maximizing the training heritability (by minimizing Eq.(9)), the resultant model may overfit the training data **X**. If overfitting occurs, the optimal **w **of Eq.(9) may correspond to a trait that has high heritability on the data that is used to train the linear model, but when the model is applied to a new sample, the trait has low heritability. In order to prevent overfitting and identify a trait with high heritability that can generalize, we incorporate a regularizer *R*(**w**) in our formulation (9). The optimization problem becomes:

(10) $$\begin{array}{cc} \;\;\;\underset{w}{\text{min}} & \frac{1}{n}w^{T}\left(X^{T}PX\right)w+\frac{\lambda }{d}R\left(w\right)\\ \text{subject to} & w^{T}\left(X^{T}QX\right)w=1, \end{array}$$

where *λ *is a hyper-parameter and needs to be tuned, and $\frac{1}{n}$and $\frac{1}{d}$are included to pre-balance the two items in the objective function. The value of *λ *can either be chosen by users according to domain knowledge or determined using a crossvalidation process as done in our experiments. According to learning theory [26], minimizing $\frac{1}{n}w^{T}\left(X^{T}PX\right)w$ corresponds to empirical risk minimization, whereas minimizing the objective in Eq.(10) corresponds to structural risk minimization that improves the generalizability of the resultant model. There are many different ways to define *R*(**w**) [23]. The *L*_2 _vector norm defined by $||w|{|}_{2}^{2}=\sum_{i}w_{i}^{2}$ is a common choice. The *L*_1 _vector norm defined by $\left||w|{|}_{1}=\sum_{i}|w_{i}\right|$ can be a better choice when model sparsity is required to select variables for use in the model. In more complicated applications where variables may be grouped and feature selection among groups is expected, a structured regularizer, such as the group lasso ${\left||w|\right|}_{2,1}=\overset{L}{\underset{K=1}{\sum }}\sqrt{\sum_{i\in G_{k}}w_{i}^{2}}$, can be used where $G_{k}$ contains the indices of variables belonging to a group *k*.

### Optimization algorithm

In this paper, we use the *L*_1 _norm penalty $||w|{|}_{1}$ to be *R*(**w**), and develop an efficient algorithm to solve the resultant optimization problem as follows:

(11) $$\begin{array}{cc} \;\;\;\underset{w}{\text{min}} & \frac{1}{n}w^{T}\left(X^{T}PX\right)w\text{ + }\frac{\lambda }{d}||w|{|}_{1}\\ \text{ subject}\;\text{to} & w^{T}\left(X^{T}QX\right)w=1. \end{array}$$

The algorithm we will describe next, although is designed for Problem (11), can be modified to solve Problem (10) that may take another form of the regularizers.

Due to the use of the ${∥w∥}_{1}$ norm, the objective function in Problem (11) is not continuously differentiable and a gradient decent type of approach cannot be applied directly. A well known strategy to overcome this obstacle is to decompose **w **into two parts: **w **= **u ***− ***v**, where both **u **and **v **are vectors of the same size as that of **w**, and all the components in **u **and **v **are required to be non-negative (i.e., **u ***≥ *0, and **v ***≥ *0). BecauseXw=Xu-Xv, we denote ***γ ***= [**u***^T ^*, **v***^T ^*]*^T ^*, **H **= [**X***, −***X**], and then we have **Xw **= **H*γ***. By the change of variables, Problem (11) can be equivalently re-written as:

(12) $$\begin{array}{ccc} \underset{\gamma }{\text{min}}\;f: & \frac{1}{n}\gamma^{T}\left(H^{T}PH\right)\gamma +\frac{\lambda }{d}\overset{2d}{\underset{i=1}{\sum }}\gamma_{i} & \\ \text{subject to }\;g_{1}: & \gamma^{T}\left({\text{H}}^{T}\text{QH}\right)\gamma -1=0\\ g_{2:e}: & \gamma \geq 0, \end{array}$$

where *f *denotes the objective function, *g*'s denote the constraints, and *e *= 2*d *+ 1, indicating the number of constraints in that group. It is straightforward to show that Eq.(12) is equivalent to Eq.(11) in the sense that at optimality **w **= **u ***− ***v **=***γ***(1 : *d*) *− **γ***(*d *+ 1 : 2*d*). When Eq.(12) reaches optimality, at least one of the two components *u_i _*and *v_i _*at ny *i*-th position of the two vectors will be 0. Otherwise, by setting ${ũ}_{i}=u_{i}-v_{i}$ and ${ṽ}_{i}=0$ if *u_i _≥ v_i_*, or ${ũ}_{i}=0$ and ${ṽ}_{i}=v_{i}-u_{i}$if *u_i _< v_i_*, we obtain a better solution with${ũ}_{i}$ and ${ṽ}_{i}$ than (**u**, **v**). Therefore, at optimality, $\overset{2d}{\underset{i=1}{\sum }}\gamma_{i}=\overset{d}{\underset{i=1}{\sum }}u_{i}+v_{i}=\overset{d}{\underset{i=1}{\sum }}|w_{i}|={∥\text{w}∥}_{1}$ Then, Eq.(12) becomes exactly the same as Eq.(11).

Eq.(12) is not a convex problem because of the quadratic equality constraint. However, it can be efficiently solved using a sequential quadratic programming (SQP) algorithm [27] because both of the objective and constraints are either in a quadratic or a linear form. The gradient of the objective and constraint functions with respect to *γ *can be calculated as:

$$\begin{array}{c} \nabla f=\frac{2}{n}\left(H^{T}PH\right)\gamma +\frac{\lambda }{d}1,\\ \nabla g_{1}=2\left(H^{T}QH\right)\gamma ,\\ \nabla g_{2:e}=I\text{.} \end{array}$$

Let ***α ***be the Lagrange multipliers, the Lagrangian function of this problem can be written as:

$$L\left(\gamma ,\alpha \right)=f\left(\gamma \right)+\underset{i}{\sum }\alpha_{i}g_{i}\left(\gamma \right);$$

and the Hessian of the Lagrangian function with respect to ***γ ***is computed as:

$$\nabla_{L}^{2}=2{\text{H}}^{T}\left(\frac{\text{P}}{n}+\alpha_{1}\text{Q}\right)\text{H}.$$

We iteratively search for the optimal solution to Eq.(12). In the *t*-th iteration, we have the iterates ***γ**_t _*and ***α**_t_*, and we first solve the following quadratic program to find the moving direction for ***γ ***and ***α***,

(13) $$\begin{array}{cc} \;\;\;\underset{p}{\text{min}} & f\left(\gamma_{t}\right)+\nabla f{\left(\gamma_{t}\right)}^{\text{T}}p+\frac{1}{2}p^{\text{T}}\nabla^{2}L\left(\gamma_{t},\alpha_{t}\right)p\\ \text{subject to} & \nabla g_{1}{\left(\gamma_{t}\right)}^{\text{T}}p+g_{1}\left(\gamma_{t}\right)=0\\ & \nabla g_{i}{\left(\gamma_{t}\right)}^{\text{T}}p+g_{i}\left(\gamma_{t}\right)≻0,i\in \left[2:e\right]. \end{array}$$

The optimal solution $\hat{p}$ to the problem (13) will give the next moving direction for ***γ***, along which the objective of Problem (12) can be decreased. Let$\hat{\text{q}}$ be the optimal Lagrange multipliers of the problem (13) corresponding to $\hat{\text{p}}$. The next moving direction of ***α ***is calculated as$\hat{\text{q}}-\alpha_{t}$. After the moving directions are computed, we then employ a line search method described in [27] to find the optimal searching step size *s *and update ***γ ***and ***α ***as follows:

(14) $${\gamma_{t}}_{+1}=\gamma_{t}+s{\hat{\text{p}}}_{t},\;{\alpha_{t}}_{+1}=\alpha_{t}+s\left({\hat{q}}_{t}-\alpha_{t}\right).$$

We summarize the proposed algorithm in Algorithm 1. It has been proved that a SQP based algorithm can converge to a local minimizer $\hat{\gamma }$ of the optimization problem (12) [28].

**Algorithm 1 **A sequential quadratic programming approach to solving Problem (11)

### Data sets

We validated the proposed approach in both simulations and the analysis of a real-world data set that was aggregated from multiple genetic studies of cocaine dependence (CD).

#### Cocaine use and related behaviors data

We used the *Semi-Structured Assessment for Drug Dependence and Alcoholism *(SSADDA) dataset aggregated from genetic studies of drug dependence to evaluate the proposed algorithm. The SSADDA subjects were recruited from multiple sites, including the University of Connecticut Health Center, Yale University School of Medicine, the University of Pennsylvania School of Medicine, McLean Hospital and the Medical University of South Carolina. All subjects participated using procedures approved by the institutional review board at each participating site. There were 6,621 subjects genotyped with a total of 1,140,420 SNPs genome-wide. Among the subjects, 2,674 were stratified into the African American population using STRUCTURE software v2.3 [29], and only these subjects were used in our experiments to avoid spurious findings due to population structure. We removed 537 subjects who had other family members in the data so the GRM was computed for unrelated individuals.

**Input: Z**, **C**, **X**, *λ*

Output: *γ*

1. Calculate **P **according to Eq.(7), and **Q **according to Eq.(8).

2. Initialize ***γ ***with **u **= **1**, **v **= **0**.

3. Initialize the Lagrange multipliers ***α ***= 1.

4. Evaluate *f*, ∇f, ∇*g_i _*and $\nabla^{\text{2}}L$with the current ***γ ***and ***α***.

5. Solve Problem (13) to obtain $\hat{\text{p}}$ and $\hat{q}$.

6. Perform a line search to find the searching step size *s*.

7. Update ***γ ***and ***α ***as in Eq.(14). Repeat 4-7 until ***γ ***reaches a fixed point.

A series of data cleaning steps were performed to ensure the quality of genotypic markers. Markers that meet any of the following conditions were excluded: low call rate (*<*98% subjects received values for the marker), G/C and A/T markers (to avoid strand issues), deviation from Hardy-Weinberg equilibrium at *p <*10*^−8^*, significant cohort calling discrepancy and being monomorphic. We also removed non-autosomal markers, so that only markers from the 22 autosomal chromosomes were used in the analysis. After these data cleaning steps, 690,864 SNPs remained. Genetic relationship was estimated for each pair of subjects by the genome-wide complex trait analysis (GCTA) software [11] using all 690,864 SNPs. We then excluded 385 subjects whose relatedness to some subjects was greater than 0.025 (corresponding to the relatedness of second cousins). The remaining sample, 1,752 subjects, was used in our analysis.

All subjects were interviewed with a computer-assisted assessment system called the SSADDA [4], which consists of survey questions designed for cocaine use and related behaviors. All subjects were reported to have used cocaine in their lifetime. The responses to those questions in the SSADDA led to the definition of thirteen important cocaine use related variables, based on which a diagnosis of CD was determined. There were seven binary variables as listed below, which represent the seven cocaine dependence criteria in DSM-IV.

• *F1 *- tolerance to cocaine;

• *F2 *- withdrawal from cocaine;

• *F3 *- using cocaine in larger amounts or over longer period than intended;

• *F4 *- persistent desire or unsuccessful efforts to cut down or control cocaine use;

• *F5 *- great amount of time spent in activities necessary to obtain, use or recover from the effects of cocaine;

• *F6 *- gave up or reduced important social, occupational, or recreational activities because of cocaine use;

• *F7 *- cocaine use despite knowledge of persistent or recurrent physical or psychological problems likely to have been caused or exacerbated by cocaine. In our experiments, positive responses to the seven variables were coded by 1 and negative responses were coded by 0. We also included six continuous variables in the analysis as listed below:

• *F8 *- number of cocaine symptom endorsed;

• *F9 *- age when first used cocaine;

• *F10 *- age when last used cocaine;

• *F11 *- age when first diagnosed with DSM-IV cocaine dependence;

• *F12 *- age when last diagnosed with DSM-IV cocaine dependence;

• *F13 *- transition time in years between the first cocaine use and the first cocaine dependence diagnosis.

All these variables were normalized to the range of [0, 1] in the analysis.

### Synthetic data

Following the same design principle used in the simulations for testing chip heritability in [8], we used the real-life genotypic data in the CD study but synthesized phenotypic data. We simulated quantitative traits based on the mixed-effect linear model shown in Eq.(1). We first synthesized a dataset that contained 5 phenotypic features, all of which were created with moderate to high heritability, and were used to form a quantitative trait of very high heritability reaching 0.8. We then added irrelevant features, which varied mainly due to covariates, to create five other simulated datasets. These datasets consisted of 10, 20, 30, 40 and 50 features where only the first 5 of them were used in the model of the final trait. These datasets were used to determine whether the proposed algorithm could identify the right features for use in the model.

To synthesize features with genetic effects, we randomly picked 2,000 of the 690,864 SNPs in the cocaine use data set and used them as the causal variants of these features. The random effect coefficient *u_j _*associated with each of the 2,000 markers was generated independently by sampling from the standard normal distribution *N *(0, 1). The residual component *ε_i _*for each individual was drawn from the normal distribution of mean 0 and variance var(**z***_i_***u**)(1*/h*^2 ^*− *1) where **z***_i _*is the *i*-th row of **Z**, var(*·*) is the sample variance of a random variable and *h*^2 ^is the heritability of the feature. To synthesize features with no genetic effects, we ignored the term **Zu **in Eq.(1) and created *ε *by randomly sampling from the standard normal distribution. To further synthesize features with fixed covariate effects, we used sex and age of the individuals in the CD study as the covariates, and arbitrarily set their effects, i.e., the *β *coefficients, to 0.2 and 0.5.

We evaluated the proposed method in two different experimental settings:

**Setting 1: **This setting assumed that there were no covariate effects in the quantitative trait. The five relevant features were simulated as follows. We used the procedure described in the above paragraph to create four features with *h*^2 ^equal to 0.2: **x**_1_*, · · · *, **x**_4_. Then we simulated the final quantitative trait **y**_1 _with *h*^2 ^= 0.8 using the same procedure. A five-entry weight vector was created with arbitrary values, such as **w **= [0.22, 0.67, 0.60, 0.30, 0.22], used in our experiments. Then, the fifth feature was directly computed as $x_{5}=\left(y_{1}-\overset{4}{\underset{i=1}{\sum }}w_{i}x_{i}\right)/w_{5}$. By simulating the data in this way, we knew that there was at least one linear combination of the five features in the data that would result in a composite trait (i.e., **y**_1_) with *h*^2 ^of 0.8. Hence, if our approach worked, it should at least find this linear combination if there was no any other one that gave even higher *h*^2^. Note that the heritability of the fifth feature had to depend on the empirical estimation, but given how it was created, there were genetic effects in this feature.

We then created 45 other features that had no genetic effects, and added a certain number of these features to the original 5 features to create 5 other datasets. Hence, there were in total 6 datasets for 1,752 subjects with 5, 10, 20, 30, 40 and 50 features. We used this set of data (i.e., the discovery set) in training to retrieve the combination models. Then we repeated the above procedure to create another independent set of data (i.e., the validation set) to validate the resultant models.

**Setting 2: **This setting assumed that the two covariates, sex and age, had fixed effects to the features and the final trait. We generated 5 features by adding fixed effects to the 5 useful features created in Setting 1. Because fixed effects do not change *h*^2 ^of a trait, we computed a composite trait **y**_2 _using the same pre-specified weight vector **w **that was used in Setting 1. We then created 45 other features with only covariate effects using the procedure described early on. Five other datasets were generated consisting of 10, 20, 30, 40 and 50 features. Note that the optimal weight vector for these datasets should have zero entries for all features except the first 5 features that were synthesized. Similarly, a discovery suite of the six datasets and another suite of them were synthesized using the same procedure for training and validation, respectively.

We estimated the chip *h*^2 ^of the features created in the synthetic datasets using GCTA software. The four features synthesized with a pre-specified *h*^2 ^= 0.2 had empirical chip *h*^2 ^values 0.2 *± *0.01 in these datasets. The chip *h*^2 ^estimate of the fifth feature was 0.57 in the discovery set and 0.6 in the validation set. Because fixed effects do not affect trait heritability, the five relevant features and the final quantitative traits had the same empirical chip *h*^2 ^in Settings 1 and 2. The features simulated with no genetic effects had *h*^2 ^estimates that ranged from 0 to 0.05, and most of these features had estimates less than 10^*−*5^.

## The proposed analyses

We first validated the proposed approach in a variety of experiments with the synthetic data. Then we applied our approach to the real-life cocaine use data to identify important components or subtypes of the disease defined by linear combinations of clinical features. Such a combination can be used to define a disease subtype because it produces a quantitative trait for each individual, which amounts to the membership likelihood of the individual in a subtype. Because the actual causal variants were known for synthetic data, we calculated the GRM of the individuals directly using the causal variants. In the case study for CD, because the real causal variants were unknown, we followed the commonly-used procedure in the literature on chip heritability estimation [30] and computed the GRM using all 690,864 SNPs that remained in the data. All of the reported chip heritability was estimated using GCTA software.

**Tuning of the hyperparameter: **For both the simulation and the CD case study, we performed 10 times three-fold cross validation (CV) to help determine a proper value of *λ*. At each fold of the CV, a linear model was derived by running the proposed method on 2/3 of the data in the dataset, and then tested on the remaining 1/3 of the data. The cross validated *h*^2 ^of the derived trait was estimated using only subjects in the remaining 1/3 of the data which was not used to train the model. We ran the same CV process for each pre-specified choice of *λ *(the choices we used are reported in the results section) and chose the *λ *value that gave a trait of the highest cross validated *h*^2 ^for each experimental setting.

**Evaluation metrics: **We reported and investigated the CV performance (including the mean values and standard deviations of the validation *h*^2 ^obtained in the CV process described above) for each *λ *choice in each experiment. Once the best value of *λ *was chosen through the cross validation for a dataset, we applied the proposed approach with the best *λ *to the entire data in the dataset to derive a quantitative trait. The chip heritability of this trait was estimated using the separately-synthesized validation datasets in simulations and by another cross validation process in the case study. In other words, in simulations, we estimated the valiation chip *h*^2 ^using the trait values computed by the linear model on the newly-synthesized validation samples. In the CD case study, we computed the trait *h*^2 ^using SNPs of 2/3 of the subjects randomly sampled from the dataset and repeated the random sampling 10 times to report the averaged *h*^2 ^value. We named this process the evaluation CV. Moreover, besides the heritability as a major evaluation metric, we also measured the effectiveness of our approach by comparing the derived trait models and the linear model implanted in the simulated data. We calculated the squared difference between the learned weights $\hat{\text{w}}$ and the true weights **w**, i.e., $\text{SE}\left(\text{w}\right)=\;||\text{w}-\hat{w}|{|}_{2}^{2}$ and the mean of squared residuals SE(y)=(1/n) ∑i=1n(yi-xiw^)2, and reported the values in plots. Additional evaluation steps were conducted in the case study to clinically interpret the resultant quantitative trait (see the later paragraph).

**Comparison: **The validated chip *h*^2 ^of our derived traits was compared with that of all quantitative features in the data in both simulations and the CD case study. In each of the experiments, our derived trait was also compared with the commonly-used disease phenotype, often referred to as a symptom count, which was the quantitative trait created by equal weighted aggregation of all features in the data. Given that no prior method existed to identify heritable disease components using the genome-wide SNPs, on the real-life cocaine use data, we compared our approach with a recently published method [23] that aimed to derive linearly-combined traits using pedigrees of related individuals. This comparison considered whether a pedigree-based heritable component analysis method can identify a disease component with a chip heritability comparable to that found by our approach. As multi-member families were included in the original cocaine use dataset, i.e., a superset of the sample used by our approach, it was feasible to apply the method in [23] to derive a trait. Then we computed the trait values on the unrelated individuals used by our approach to compare the chip *h*^2 ^of the two approaches using the evaluation CV. Note that the prior pedigree-based approach was actually given a favor because it used the superset of 2,674 subjects (unrelated individuals were treated as one-member pedigrees) to derive the trait in comparison with our approach that used only the 1,752 unrelated individuals.

**Clinical interpretation: **It is very important to understand the clinical implications of the quantitative trait (or an empirical subtype) derived by our approach from the aggregated CD study data. From prior work [17,31], we identified three key steps to ensure the clinical validity of an empirical subtype. We first examined the specific features selected by our approach for use in the model. Second, we studied the distribution (or histogram) of the quantitative scores among the 1,752 subjects. From the distribution plot, we examined whether there were obvious subgroups of the scores. Third, the subgroups of subjects were characterized and compared on 11 of the most important clinical variables reflecting cocaine use and related behaviors including both the features selected and those not selected for use in the linear model. The individuals receiving very high or very low values of the quantitative trait may show the most representative features of the subtype.

## Results

### Simulations

**Figure 1:**
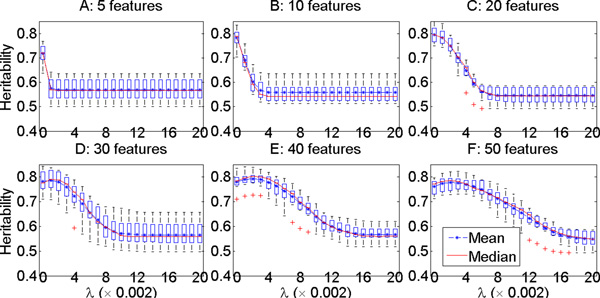
**Simulation study: the **testing ***h*^2 ^of the quantitative traits developed in three-fold cross validation with varying *λ *and total number of phenotypic features in setting 1, in which datasets are simulated without covariate effect on the phenotypic features**.

**Figure 2:**
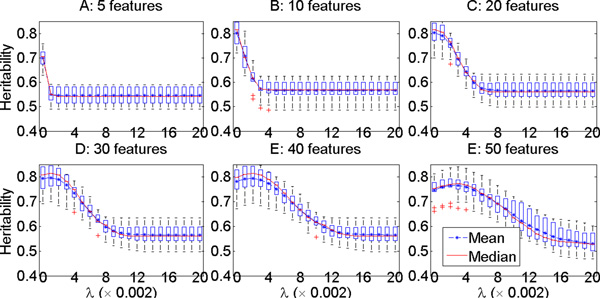
**Simulation study: the **testing ***h*^2 ^of the quantitative traits developed in three-fold cross validation with varying *λ *and total number of phenotypic features in setting 2, in which datasets are simulated with covariate effect on the phenotypic features**.

We pre-specified 21 different *λ *values ranging from 0 to 0.04 with step size 0.002 for use in the cross-validation tuning process. The validation or test *h*^2 ^for each *λ *choice was plotted for each of the six datasets in Figure 1 (for Setting 1 where data were generated without covariate effects) and Figure 2 (for Setting 2 where data were generated with covariate effects). The mean, median, and the standard deviations of the test *h*^2 ^values in the cross validation were plotted for each tested *λ*. These two figures show that our approach could identify components (quantitative traits) with test *h*^2 ^estimate of ~ 0.8, which was the heritability of the implanted heritable component (the simulated true model), for all datasets even with many irrelevant features in some of the datasets for both settings. This result demonstrates that our approach identified highly heritable disease components and could successfully correct for fixed covariate effects.

In addition, both Figures 1 and 2 show that there was overfitting of the learned models to the training data when *λ *was too small, especially when the number of irrelevant features grew to 25, 35 and 45. The larger the number of irrelevant features in the data, the more severe was the overfitting seen when *λ *was small. On the other hand, when *λ *was too large, underfitting could occur, so the test *h*^2 ^showed a peak in most of the plotted curves. The best choice of *λ *for the six datasets (sorted from the smallest number of features in the data to the largest number of features) were 0, 0, 0, 0.002, 0.004 and 0.004 for Setting 1, and 0, 0, 0, 0,002, 0.004 and 0.006 for Setting 2.

**Figure 3:**
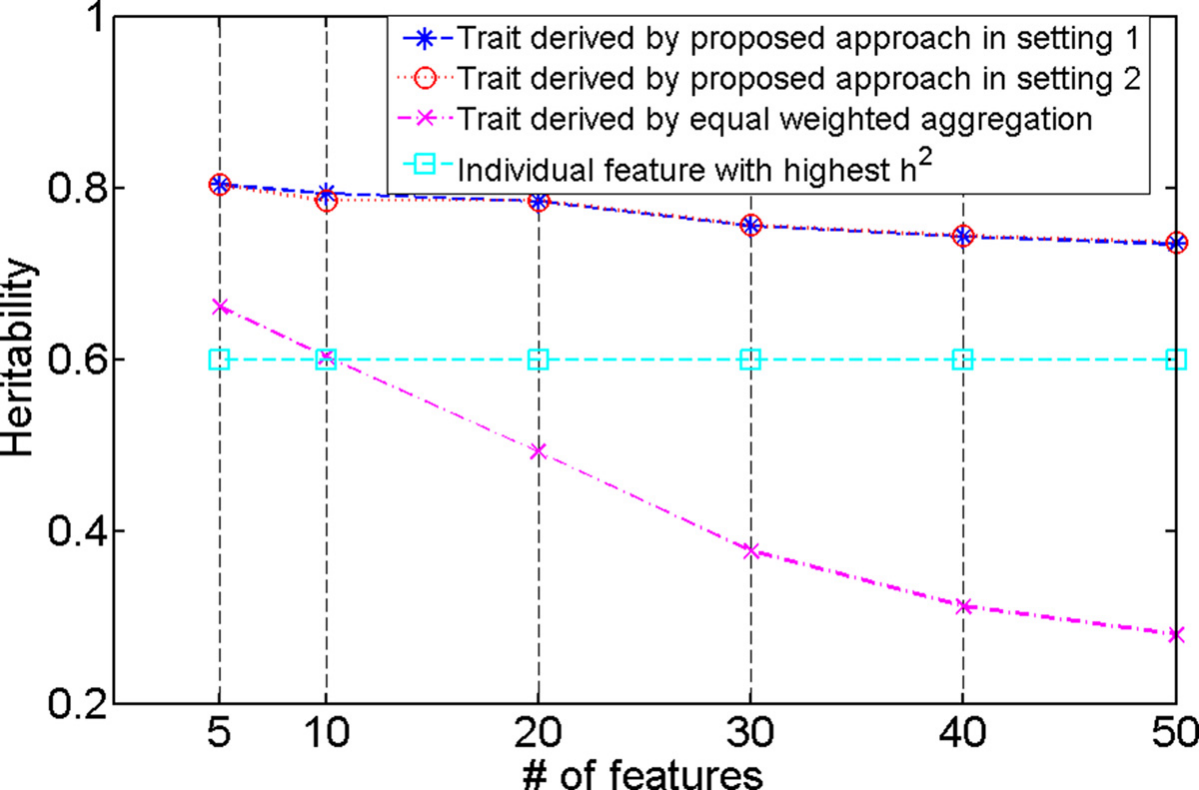
**Simulation study: comparison of *h*^2 ^of commonly used traits with that of the quantitative traits derived by the proposed approach with chosen *λ*'s**. Results from both settings (setting 1 - without covariates, setting 2 - with covariates) are shown. For *h*^2 ^of individual features in each dataset, we show the highest. Because in our simulation the feature that has the highest *h*^2 ^estimate is shared across all the datasets with varying number of features, the corresponding *h*^2 ^curve is a straight line. Since covariates do not affect the *h*^2 ^estimate when their effect is properly corrected for during the estimation, we do not differentiate the two settings and show one curve for traits derived by equal weighted aggregation of individual features in the data and one for individual feature that has the highest *h*^2 ^estimate.

**Figure 4:**
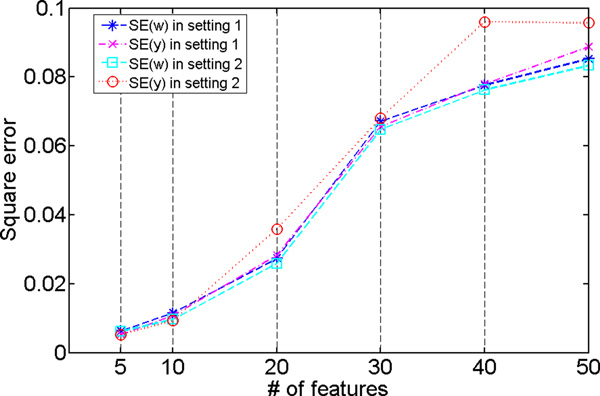
**Simulation study: the square error of feature weights (**w**) in models derived by the proposed approach with chosen *λ*'s: SE(**w**) and that of resulted quantitative traits (**y**): SE(**y**), comparing to the optimal (implanted) model coefficients $\left(\hat{w}\right)$and traits $\left(\hat{\text{y}}\right)$**. SE(**w**) is calculated as $||\text{w}-\hat{w}|{|}_{2}^{2}$; and SE(**y**) is calculated as $||\text{y}-\hat{y}|{|}_{2}^{2}/n$, where *n *is the total number of subjects in 2 2 the data. Results from both settings (setting 1 - without covariates, setting 2 - with covariates) are shown.

Then *λ *was set to the optimal value for each of the six datasets, and we re-ran the algorithm to generate the final quantitative trait from each dataset. We then compared the chip *h*^2 ^between these derived traits and the commonly used disease traits, such as individual features and the equally weighted aggregation of individual features. The results are shown in Figure 3 where all *h*^2 ^values were estimated using only the *validation *datasets. Results from both settings are shown. For all the datasets and for both settings, our approach could recover the quantitative traits of *h*^2 ^close to 0.8. When the number of irrelevant features in the data increased, the *h*^2 ^values of the derived traits decreased as expected. Typically, when more irrelevant features were included in the data, the learning problem became more challenging. Because covariates do not affect *h*^2 ^estimate when their effect is properly corrected during the estimation, we did not differentiate the two settings when discussing and comparing the *h*^2 ^values of individual features and the aggregated traits. Recall that in our simulation, the highest chip *h*^2 ^of individual features was 0.6 in validation. Figure 3 also included this most heritable feature for comparison. Among the traits derived by equally weighted aggregation, the one developed from the 5-feature dataset reached the highest *h*^2 ^(= 0.66). As expected, chip *h*^2 ^of these traits decreased along with the increasing number of irrelevant features. The trait developed from the 50-feature dataset had the lowest *h*^2 ^(= 0.28). These results demonstrate that our approach could identify quantitative traits that are more heritable (i.e., with high chip heritability) than those commonly used. The squared difference (error) between learned weights and the optimal (implanted) weights: SE(**w**) together with the squared error between the derived traits and simulated traits: SE(**y**) are presented in Figure 4. The results from both settings are provided. We observed that clearly when the number of irrelevant features increased, the noisier data made the learning problem more difficult, and SE(**y**) and SE(**w**) increased in both of the settings.

### A case study of cocaine dependence

**Figure 5:**
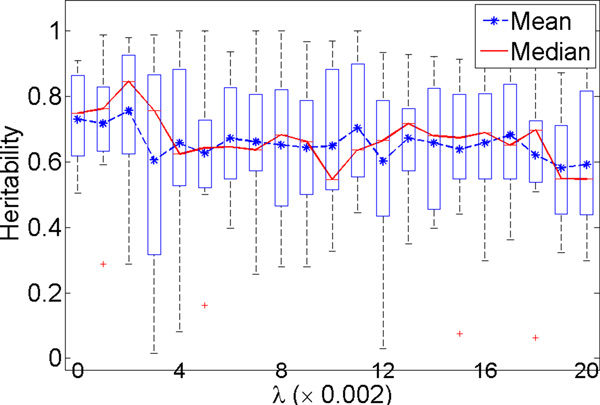
**Case study on cocaine dataset: the testing *h*^2 ^of the composite traits derived in three-fold cross validation with varying *λ***.

In this study, we used the same pre-specified *λ *values in the simulations. In all expriments, we used age, sex and the first three principal components of the GRM as covariates. The test *h*^2^s of all traits derived for each *λ *choice in the CV process are plotted in Figure 5. It shows that there was overfitting when *λ *was too small as well. The trait derived with *λ *= 0.004 achieved the highest cross-validated *h*^2 ^on average. We thus derived a model by running our approach with *λ *= 0.004 and all the 1,752 subjects in the data. We examined this model and the resultant quantitative trait as discussed in the proposed analyses section.

**Figure 6:**
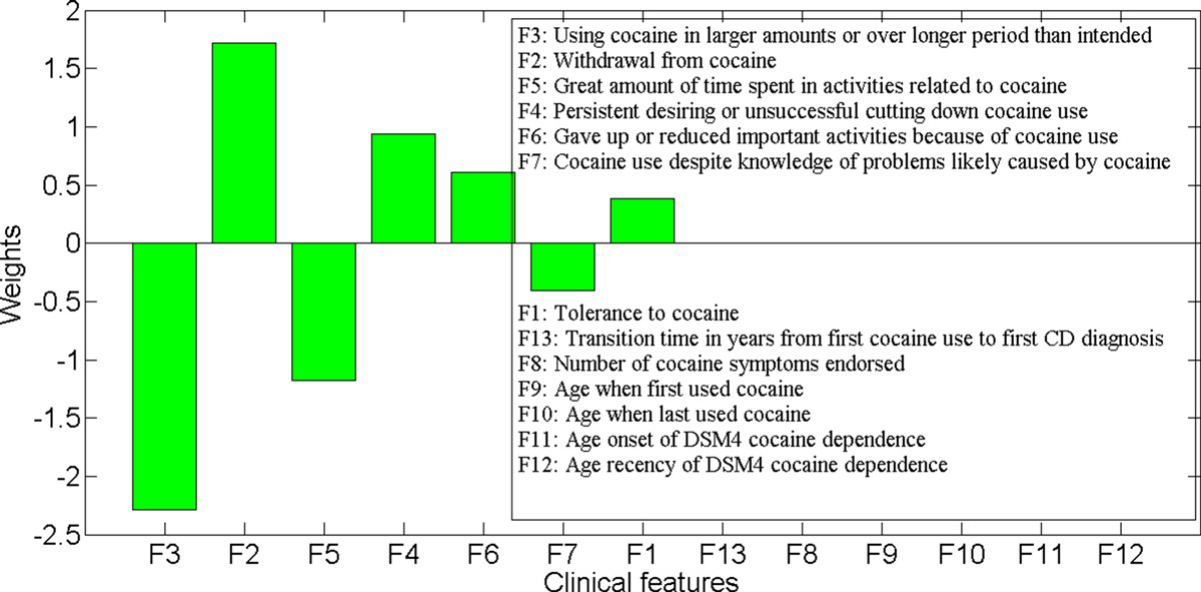
**Case study on cocaine dataset: the weights of variables in the linear combination learned by the proposed method with *λ *= 4 and the entire sample set**.

The weights that each variable received in the model are shown in Figure 6. Of the 13 clinical variables, five (F8 - F12) received a zero coefficient, and were completely ruled out from the model. Variable F13 had an coefficient close to 0 (< 10^*−*5^), thus its impact on the resultant trait was minimal. Variables with significant coefficients in the model are the seven cocaine criteria: F3 - using cocaine in larger amounts or over longer period than intended, F2 - withdrawal from cocaine, and F5 - great amount of time spent in activities necessary to obtain, use or recover from the effects of cocaine, had the highest impact on the trait. Both F3 and F5 had negative weights, which indicated that positive response to these two variables would lower the score or value of this trait. In contrast, F2 had a positive coefficient, which indicated that a positive response to this variable would increase the score. Variables F4 - persistent desire or unsuccessful efforts to cut down or control cocaine use, and F1 - tolerance to cocaine, had limited impact on the trait.

**Figure 7:**
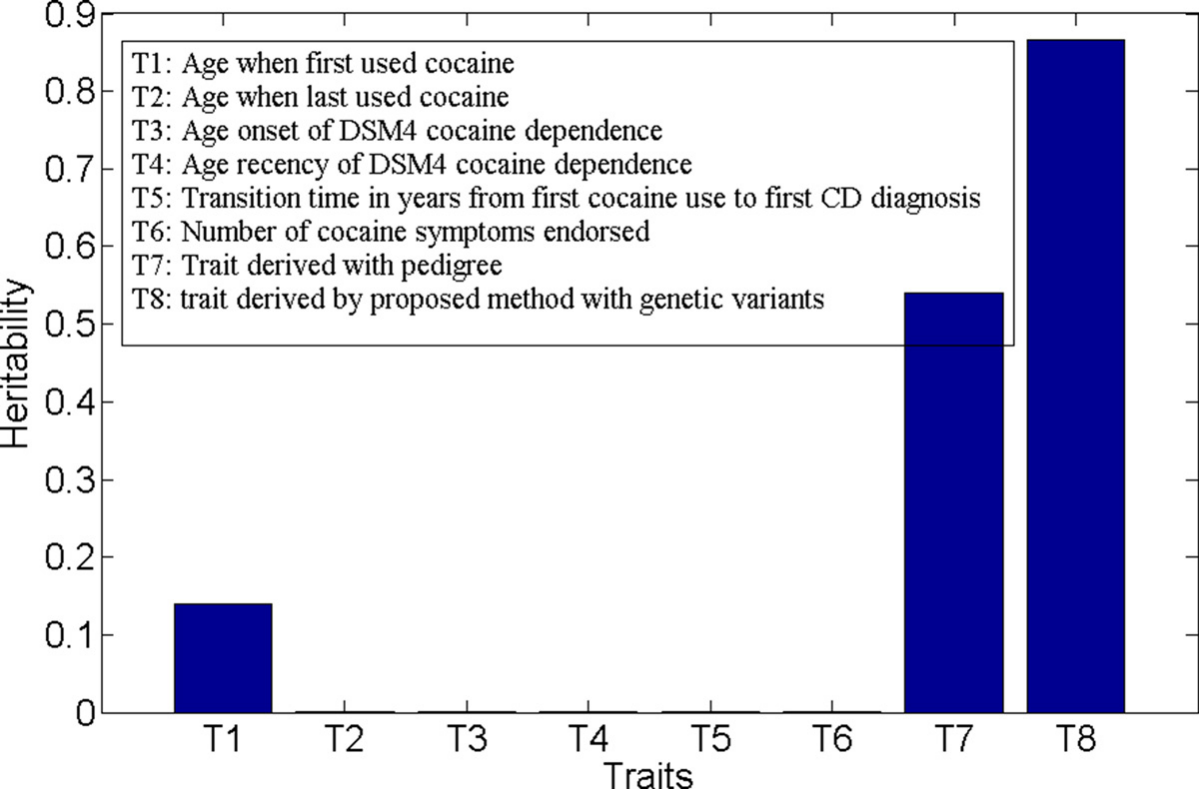
**Case study on cocaine dataset: comparison of *h*^2^'s of six individual continuous variables in the data, composite traits derived by the approach in **[23]**and that derived by the proposed approach with *λ *= 2**.

The cross validated *h*^2 ^estimate of the trait derived by the proposed approach is 0.87 (with a standard error of 0.13). For comparison, we ran the approach proposed in [23], which identifies heritable components with pedigrees as genetic inputs and its formulation also had a hyper-parameter *λ*. For fair comparison, we first ran 10 times cross validation to choose a proper value for *λ*. With this *λ*, we developed a trait using the entire African American sample set. We then estimated the trait *h*^2 ^using the exact same setting (i.e., GRM and covariates) as for the traits derived by the proposed approach. We estimated the *h*^2 ^for all six continuous variables in the data using the same setting. All of the *h*^2 ^values are plotted in Figure 7 together with the trait derived by the proposed approach. The figure clearly shows that the trait derived by the proposed approach had the highest *h*^2 ^among all compared traits, and was significantly higher than that of the trait derived using the prior approach [23]. Note that one of the continuous variables in the cocaine use data was the counting of CD criteria. It was defined as the number of positive responses to the seven CD criteria, i.e., a quantitative trait resulting from a linear combination of F1-F7 with equal weights. This trait was reported to be a better trait for genomewide association analysis than the binary CD diagnosis [1]. The CD diagnosis had a value of *h*^2 ^close to zero when estimated using our data. These results demonstrate the effectiveness of our approach in identifying disease components with high chip heritability from complex multivariate phenotypes. It is worth noting that the trait with the second highest *h*^2 ^estimate was the one derived using the prior approach [23]. This implies that (1) both of these two methods (i.e., the proposed method and the one previously reported [23]) can identify heritable components with high chip heritability; but (2) the proposed method outperforms as it directly maximizes heritability using genetic variants.

**Figure 8:**
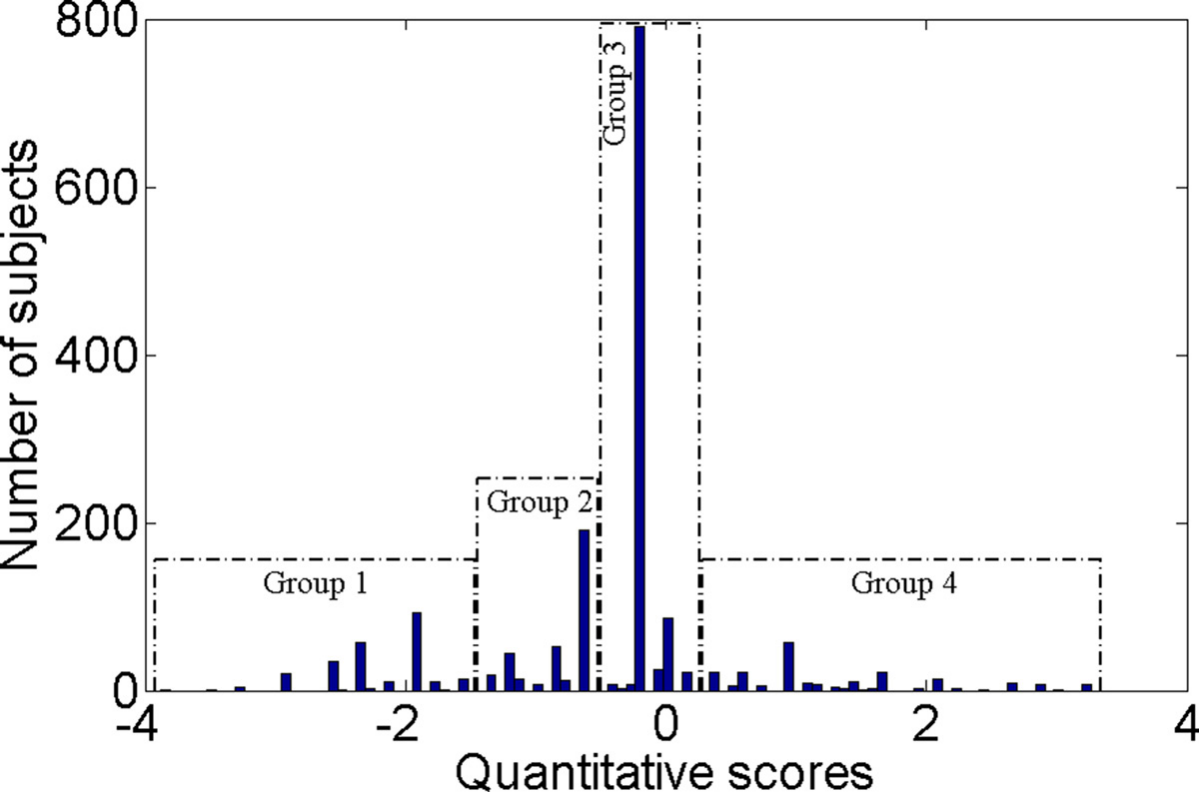
**Case study on cocaine dataset: the distribution of scores for the composite trait derived by the proposed method with *λ *= 2 and the entire sample set**.

Figure 8 shows the distribution of the trait values (i.e., the membership scores) of the subjects. It shows that based on the scores, the samples can be partitioned into four subgroups. There were 250 subjects (14.27% of total) in Group 1, which had a mean score of -2.22. Group 2 consisted of 323 subjects and comprised 18.44% of the entire sample set. Its mean score was -0.8. Group 3 was the largest one and consisted of 821 subjects (46.86% of the sample). The mean score of this group was -0.2. Group 4 was the smallest, comprised of 237 individuals (13.53% of the sample), with a mean score of 1.22.

To understand the clinical implications of the derived trait, we characterized the four groups using 11 important clinical variables, including the 7 CD criteria (F1-F7), the total number of CD criteria endorsed (F8), age of first cocaine use (F9), age when first diagnosed with DSM-IV CD (F11), and the transition time in years from first cocaine use to first DSM-IV CD diagnosis (F13). The results are summarized in Table 1. Only 5.6% of the subjects in Group 1 had experienced cocaine withdrawal symptoms (F2), despite the face that this group contained heavy cocaine users (as shown by other variables). Most of the subjects (99.6%) in this group reported using cocaine in larger amounts or over a longer period than indended (F3). Moreover, this group had the highest percentage of subjects (96%) who spent a great amount of time in activities related to cocaine (F5). Group 2 had the lowest percentage of subjects (23.6%) with tolerance to cocaine (F1), but the highest percentage of subjects (92.92%) who used cocaine despite knowledge that problems were likely caused by cocaine (F7). Subjects in Group 3 endorsed all of the seven CD criteria with similar percentages (87.04% was the lowest and 90.60% was the highest among the seven criteria). Group 4 had the lowest percentage of subjects who endorsed F3, F5 and F7. Group 4 also had the highest percentage of subjects with persistent desire or unsuccessful attempts to cut down their cocaine use (F4). Group 1 and Group 2 had a similar transition time from first cocaine use to first CD diagnosis (F13): 8.01 years for Group 1 and 8.07 years for Group 2. These were significantly shorter than the transition time in Groups 3 and 4, which were 12.15 years and 15.19 years, respectively.

**Table 1:** Characteristic of the three subject groups on important clinical variables related to cocaine use.

Variable	Group1 250(14.27)	Group2 339(19.35)	Group3 926(52.85)	Group4 237(13.53)
Tolerance to cocaine	124(49.60)	80(23.60)	807(87.15)	123(51.90)

Withdrawal from cocaine	14(5.60)	275(81.12)	813(87.80)	192(81.01)

Using cocaine in larger amounts or over longer period than intended	249(99.60)	323(95.28)	816(88.12)	103(43.46)

Persistent desiring or unsuccessful cutting down cocaine use	223(89.20)	326(96.17)	839(90.60)	233(98.31)

Great amount of time spent in activities related to cocaine	240(96.00)	290(85.55)	823(88.88)	82(34.60)

Gave up or reduced important activities because of cocaine use	170(68.00)	212(62.54)	806(87.04)	156(65.82)

Cocaine use despite knowledge of problems likely caused by cocaine	209(83.60)	315(92.92)	817(88.23)	170(71.73)

Number of CD criteria endorsed	4.92(1.07)	5.37(1.08)	6.18(2.10)	4.47(1.50)

Age when first used cocaine	21.93(6.34)	22.32(6.51)	21.44(5.75)	22.97(8.42)

Age onset of DSM4 cocaine dependence	28.24(7.88)	28.31(7.95)	26.63(6.70)	28.32(8.06)

Transition time in years from first cocaine use to first CD diagnosis	8.01(12.05)	8.07(12.91)	12.15(21.30)	15.19(24.65)

*N *(%) is shown for the first seven binary variables, where *N *is the number of subjects who are positive on the corresponding variable within a group and % is the percentage of *N *in the group.

*µ*(*σ*^2^) is shown for the last four continuous variable, where *µ *is the group mean and *σ*^2 ^the standard deviation.

## Conclusion

We developed an approach to identify composite traits from multivariate phenotypes that are highly heritable, as estimated using genome-wide SNPs. The trait we derived is in the form of a linear combination of variables related to the phenotype, that is **y **= **Xw**. A quadratic optimization problem was formulated, in which optimal **w **was sought to optimize the log likelihood for estimating variance components in REML. In this formulation, variance components are set to their ideal values with the additive genetic variance component $\sigma_{g}^{2}$ equal to 1 and other components equal to 0. To avoid overfitting, we incorporated a regularization term in our formulation. An efficient algorithm based on the sequential quadratic programming framework was developed to solve the proposed optimization problem. We evaluated the proposed approach on both synthetic and real world data. The empirical results demonstrate the effectiveness of our approach as a means to identify traits with much higher chip *h*^2 ^than commonly-used disease phenotypes.

In this paper, the pairwise genetic relationship among subjects was estimated from genome-wide SNPs. However, it can also be estimated from SNPs restricted to a specific region, such as on a particular chromosome or in genes related to a pathway, to explore the genetic architecture of a trait. When SNPs within a specific region are used, the trait resulting from the proposed approach will achieve the maximized genetic variance component corresponding to this region. In an application, such as substance dependence, there are known pathways involved, so it may be of utility to determine whether there is a composite trait, the variance of which can be largely explained by the variants within the pathways. This will be a future application of our approach.

## Competing interests

The authors Jiangwen Sun and Jinbo Bi declare that they have no competing interests. Henry R Kranzler has served as a consultant or advisory board member for the following companies: Alkermes, Lilly, Lundbeck, Otsuka, and Pfizer. He is a member of the Alcohol Clinical Trials Group of the American Society of Clinical Psychopharmacology, which is supported by Abbvie, Alkermes, Ethypharm, Lilly, Lundbeck, and Pfizer.

## Authors' contributions

JB, HRK and JS designed the overall study of finding heritable disease subtypes. JS and JB designed the new algorithm discussed in this paper. JS implemented the algorithm and performed the data analyses. HRK provided the aggregated drug dependence data and helped in interpreting the results. JS and JB wrote the first draft of this manuscript.
